# Intergeneric and interspecific relationships in tribe Ricineae revealed by phylogenomics of the plastome and transcriptome

**DOI:** 10.3389/fpls.2025.1544247

**Published:** 2025-05-01

**Authors:** Wen-Xiang Liu, Guo-Bo Li, Zhuo Zhou, Jia-Fu Chen, An-Min Yu, Ai-Zhong Liu, Bin Tian, Jun-Wei Ye

**Affiliations:** ^1^ National Plateau Wetlands Research Center, Southwest Forestry University, Kunming, China; ^2^ Key Laboratory for Forest Resources Conservation and Utilization in the Southwest Mountains of China, Ministry of Education, Southwest Forestry University, Kunming, China; ^3^ Key Laboratory for Plant Diversity and Biogeography of East Asia, Kunming Institute of Botany, Chinese Academy of Sciences, Kunming, China

**Keywords:** *Discocleidion*, diversification, Euphorbiaceae, phylogeny, *Speranskia*

## Abstract

**Introduction:**

The taxonomy of Euphorbiaceae is extremely difficult, especially the phylogeny of closely related genera. In *Ricinus*, which embraces an important non-food oil-seed crop worldwide, *Discocleidion* and *Speranskia* are closely related genera based on molecular evidence (tribe Ricineae), however the intergeneric and interspecific relationship of the tribe is not well-resolved.

**Methods:**

Plastome and transcriptome were sequenced and assembled before maximum likelihood and Bayesian inference phylogenetic trees were reconstructed. Plastome features and comparative analyses were conducted. Morphological traits of the tribe were explored as supplement to the molecular data.

**Results:**

The newly sequenced plastomes ranged from 167,327 to 190,093 bp with typical circular quadripartite structures. The longest genome of *S. tuberculata* may due to higher number of simple sequence repeats. Natural selection pressure on chloroplast genes was relatively small and the tribe likely experienced a population contraction. The transcriptome assembly contig N50 of the tribe ranged from 1506 (*D. rufescens*) to 2489 bp (*S. tuberculata*). A total of 50,513 genes (*S. cantonensis*) to 78,048 genes (*D. ulmifolium*) were detected, and the GC content varied between 38.17% (*S. cantonensis*) and 40.01% (*R. communis*). The three genera formed a well-supported monophyletic lineage, confirmed by different genomic data using different methods. *Discocleidion* and *Ricinus* were supported to be closely related. In *Speranskia*, *S. yunnanensis* diverged first and the divergence of *S. tuberculata* and *S. cantonensis* was followed. Further, morphological similarities supported the monophyletic lineage and intergeneric and interspecific relationship.

**Discussion:**

The relationship in the tribe Ricineae is clearly revealed by genomic and morphological data, providing a genetic basis for future comparative genomic investigations and phylogeny reconstruction of Euphorbiaceae.

## 1 Introduction

The taxonomy of Euphorbiaceae, which includes 299 genera with *ca*. 6,500 species, is one of the most complicated among angiosperms (Govaerts et al., 2000; Sun et al., 2016). Discordance lies between/within morphological and molecular phylogenetic classifications (Webster, 2014; Sun et al., 2016). *Ricinus communis* L. is an important non-food oil-seed crop worldwide that produces rich ricinoleic acid that has been widely used in industry and its seeds contain the extremely toxic protein ricin that has been used as an immunotoxin for therapeutic purposes in different cancers (Xu et al., 2021). Unclear phylogenetically closely related genera with the *Ricinus* L. complicate the comparative genomics researches that will deepen the understanding of the formation mechanism and biochemical process of these substances.

Morphologically, *Ricinus* was first defined as the sole genus of the tribe Ricineae Bartl (Bartling, 1830) (Ord. Nat. Pl.: 371. Sep 1830.). Hutchinson (1969) merged *Homonoia* Lour., *Spathiostemon* Blume, and *Lasiococca* Hook. f. into the tribe Ricineae. Hans (1973) suggested *Ricinus* belongs to the tribe Crotoneae and the tribe Ricineae was not retained. Webster (1994) defined *Ricinus* as the single genus of the subtribe Ricininae of tribe Acalypheae and the genus appeared to be most closely related to *Adriana* Gaud. Then, the two genera were classified as the tribe Ricineae by Webster (2014). Flora of China (FOC) included *Ricinus* and *Discocleidion* (Muell. Arg.) Pax et Hoffm. in the tribe Acalypheae while genera composition was different compare to Webster (1994). *Speranskia* Baill. (a closely related genus based on molecular phylogeny, see below) was not included in Hutchinson (1969) and Hans (1973), while Webster (2014) suggested it belonged to tribe Chrozophoreae as in the FOC.

Molecular phylogenies show different relationships. Through two chloroplast fragments (*rbcL* and *trnL-F*), Wurdack et al. (2005) indicated *Speranskia* was closely related to *Ricinus* while the posterior probability (PP) was low. A similar relationship was further supported by Zhou et al. (2017) through combined 18S and four chloroplast fragments (*atpB*, *matK, rbcL*, and *trnL-F*), while *Ricinus* and *Discocleidion* were found to be more closely related when using three chloroplast fragments (except *atpB*). A close relationship between *Ricinus* and *Discocleidion* was also reconstructed through chloroplasts *rbcL*, *atpB*, and *matK* and mitochondrial *matR* gene (Sun et al., 2016); or a standardized set of 353 nuclear genes (Zuntini et al., 2024). *Speranskia* and *Discocleidion* are likely closely affiliated with *Ricinus* (the three genera are treated as tribe Ricineae in this study), however, the intergeneric relationship is inconsistent in different studies.

In addition to the inconsistencies in phylogenetic relationships arising from different fragments within the plastome, the phenomenon of cytoplasmic-nuclear conflict is also widely observed in systematic studies (Fu et al., 2023; Koenen et al., 2020; Meng et al., 2021). The discrepancy can be attributed to incomplete lineage sorting (ILS), hybridization, or introgression (Meleshko et al., 2021; Osuna-Mascaró et al., 2023). In order to clarify the intergeneric and interspecific relationships of the tribe Ricineae, both organelle (plastome) and nuclear (transcriptome) genomes were used to provide further evidence of the phylogenetic relationship in this study. The specific aims of this study were to ascertain (1) whether *Ricinus, Discocleidion*, and *Speranskia* form a well-supported monophyletic lineage and whether cytoplasmic-nuclear discordance exists for intergeneric and interspecific relationships, and (2) what are the comparative features of the plastome among different species in the tribe.

## 2 Materials and methods

### 2.1 Sample collection, sequencing, and genome assembly

**Figure 1:**
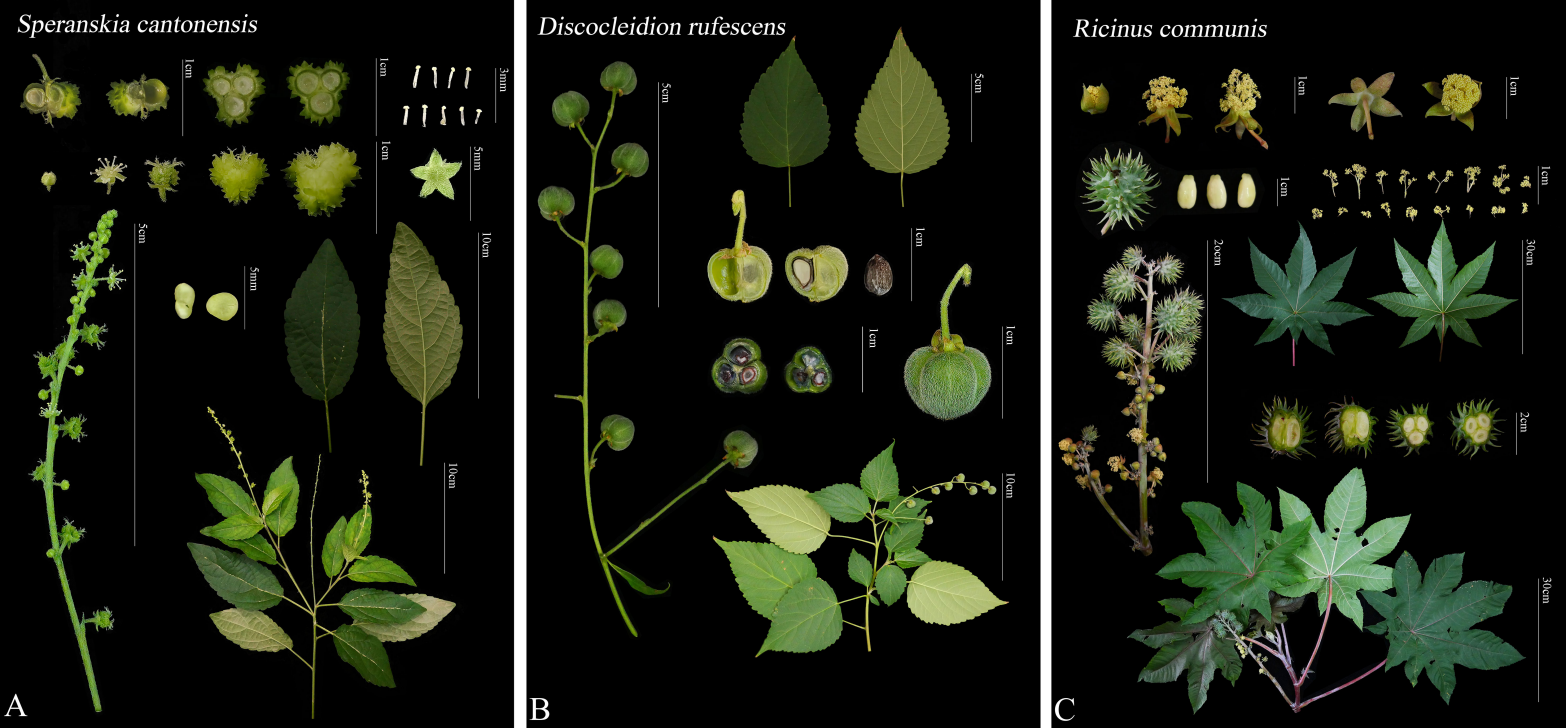
Comparison of morphological characteristics among different genera of the tribe Ricineae. **(A)** *Speranskia cantonensis*, **(B)** *Discocleidion rufescens* (female plant), **(C)** *Ricinus communis*.

Fresh leaves of *S. tuberculata*, *S. yunnanensis*, *S. cantonensis*, *D. rufescens*, and *D. ulmifolium*, representing all species in the tribe except *R. communis*, were sampled and preserved with silica gel before DNA extraction. Mixed samples of roots, stems, leaves, and flowers were sampled and preserved with liquid nitrogen for RNA extraction. Voucher specimens were kept in the herbarium of Southwest Forestry University (**Figure 1**; **Supplementary Table S1**). DNA was extracted using the Cetyltrimethylammonium bromide (CTAB) method before it was sequenced using the Illumina HiSeq 2500 platform (USA, California) by Commission Feisha Bioinformatics Co., Ltd. (Wuhan, China). RNA was extracted using an RNAsimple Total RNA Extraction Kit (DP419), sequenced on the Illumina HiSeqTM platform (USA, California), and were commissioned to NextOmics Biosciences Co., Ltd. (Wuhan, China). The raw data were filtered and cleaned using fastp v0.20.0 (Chen et al., 2018) to obtain clean reads.

The plastome was assembled using GetOrganelle v1.7.5 (Jin et al., 2020) and annotated and visualized using the online software CPGAVAS2 (Shi et al., 2019) (http://47.96.249.172:16019/analyzer/annotate) using *Nicotiana tabacum* as the reference (Kunnimalaiyaan and Nielsen, 1997). RNA-seq data of six Euphorbiaceae species (*R. communis*, *Jatropha curcas*, *Euphorbia lathyris*, *Hevea brasiliensis*, *Manihot esculenta*, and *Mercurialis annua*) and *Arabidopsis thaliana* were downloaded from GenBank (**Supplementary Table S2**). The quality of the downloaded data was assessed using FastQC (https://www.bioinformatics.babraham.ac.uk/projects/fastqc/) to identify low-quality sequences and adapter contamination. Unstable sequences were subsequently removed with fastp v0.20.0 (Chen et al., 2018). The filtered data were assembled using Trinity v2.91 (Garber et al., 2011). To reduce redundancy in the assembly results, CD-HIT was employed. Finally, open reading frames (ORFs) and predicted coding sequences were identified using TransDecoder (Li and Godzik, 2006).

### 2.2 Plastome features and comparative analyses

#### 2.2.1 Selective pressure analyses

DNAsp5 (Librado and Rozas, 2009) was used to calculate the Ka/Ks value and non-synonymous substitution, and a Ka/Ks heatmap was plotted using ChiPlot (https://www.chiplot.online). A neutrality test inferred using Tajima’s D was also tested in DNAsp5.

#### 2.2.2 Similarity analyses

Nucleotide diversity index (Pi) was calculated using DNAsp5 (Librado and Rozas, 2009). CPStools (Huang et al., 2024) was used to evaluate the Pi of the coding and non-coding regions. Then, hypervariable regions with high Pi values were selected as the candidate areas for allele-specific barcodes. Using *Mallotus japonicus* (JF937588.1), a close relative species in Euphorbiaceae, as a reference, the plastome similarity of the six species was analyzed using mVISTA (Frazer et al., 2004) and the Shuffle-LAGAN under the condition of detecting rearrangements by performing global pairwise alignments of sequences. A similar analysis was also performed among species from different clades within the Subfam. Acalyphoideae and the tribe Ricineae. IRscope (Amiryousefi et al., 2018) was used to compare the boundaries and contractions or expansions of the plastome inverted repeat (IR) regions. Circoletto (Chen et al., 2022) was used to conduct collinearity analysis on the plastomes.

#### 2.2.3 Codon usage analysis

Codonw (Peden, 2000) was used to analyze the third-position base content (A, T, C, and G) of the codons and calculate the codon adaptation index (CAI), codon bias index (CBI), effective number of codons (ENC), and frequency of optimal codons (Fop). The CAI module in Python was used to compute the relative synonymous codon usage (RSCU) and the ggplot2 package in R 3.4.1 (https://www.r-project.org/) was used for data visualization.

#### 2.2.4 Repeat sequences and simple sequence repeats (SSRs)

First, large segment repetitive sequences (length ≥ 30 bp, Hamming distance = 3) were annotated using REPuter (Kurtz et al., 2001), including forward (F), reverse (R), complement (C), and palindromic (P) patterns. Then, MISA (Beier et al., 2017) was used for SSRs analysis.

### 2.3 Phylogeny and divergence time estimations

The six plastomes, along with the published plastomes of other Euphorbiaceae species, were aligned and manually adjusted using MAFFT (Katoh et al., 2002). A maximum likelihood (ML) tree was reconstructed in IQ-TREE (Nguyen et al., 2015) using GTR+F+R3 which is the best model selected using IQ-TREE after 1,000 iterations. A Bayesian inference tree (BI) was created in MrBayes (Huelsenbeck and Ronquist, 2001). After performing the variance test, the HKY model was selected. We conducted divergence time estimations using two calibration points. The fossil *Hippomanoidea warmanensis* (Crepet and Daghlian, 1982) was assigned to the origin node of the Subfam. Acalyphoideae, with a minimum time set at 43 million years ago (Ma) (Anest et al., 2021). Additionally, a secondary calibration was applied to the divergence time between *A. thaliana* and *E. lathyris* which was set to 108 ± 4.0 Ma (Lu et al., 2022). FigTree (http://tree.bio.ed.ac.uk/software/figtree/) was used to visualize all phylogenetic trees.

In the transcriptome data, orthologous genes were identified using the Blast function in Orthofinder v2.5.4 (Emms and Kelly, 2019). These orthologous genes were then clustered to form orthogroups, and gene trees were constructed for each gene family. Subsequently, the gene trees were merged into a species tree using a concatenation method. A total of 192 single-copy orthologous genes were aligned and modified using trimAl v1.4 (Capella-Gutiérrez et al., 2009). Gaps were removed to ensure that the filtered sequences accounted for no less than 70% of the original sequences. A ML phylogenetic tree was reconstructed using RAxML v8.2.12 (Stamatakis, 2014) with the PROTGAMMAJTT model. Divergence times between *A. thaliana* and *E. lathyris* (108 ± 4.0 Ma) and between *E. lathyris* and *J. curcas* (62 ± 1.0 Ma) obtained from the TimeTree (http://www.timetree.org) were used as two secondary calibrations in the MCMCtree method implemented in the PAML v4.0 (Yang, 2007).

### 2.4 Morphological analysis

The morphological traits of the three genera (*Ricinus*, *Discocleidion*, and *Speranskia*), along with their easily confused taxa, were analyzed through a combination of field observations, collection of live specimens and herbarium samples, and morphological descriptions were sourced from FOC.

## 3 Results

### 3.1 Transcriptome features

The transcriptome assembly contig N50 of the six species within the tribe Ricineae ranged from 1,506 bp (*D. rufescens*) to 2,489 bp (*S. tuberculata*), with an average contig length of 802 bp (*D. rufescens*) to 1,923 bp (*R. communis*). A total of 50,513 genes (*S. cantonensis*) to 78,048 genes (*D. ulmifolium*) were detected, and the GC content varied between 38.17% (*S. cantonensis*) and 40.01% (*R. communis*). The completeness ranged from 93% for *D. rufescens* to 95.5% for *R. communis*, with an average of 95.9% (**Supplementary Table S3**), indicating high assembly completeness.

### 3.2 Plastome features and comparative analyses

**Figure 2:**
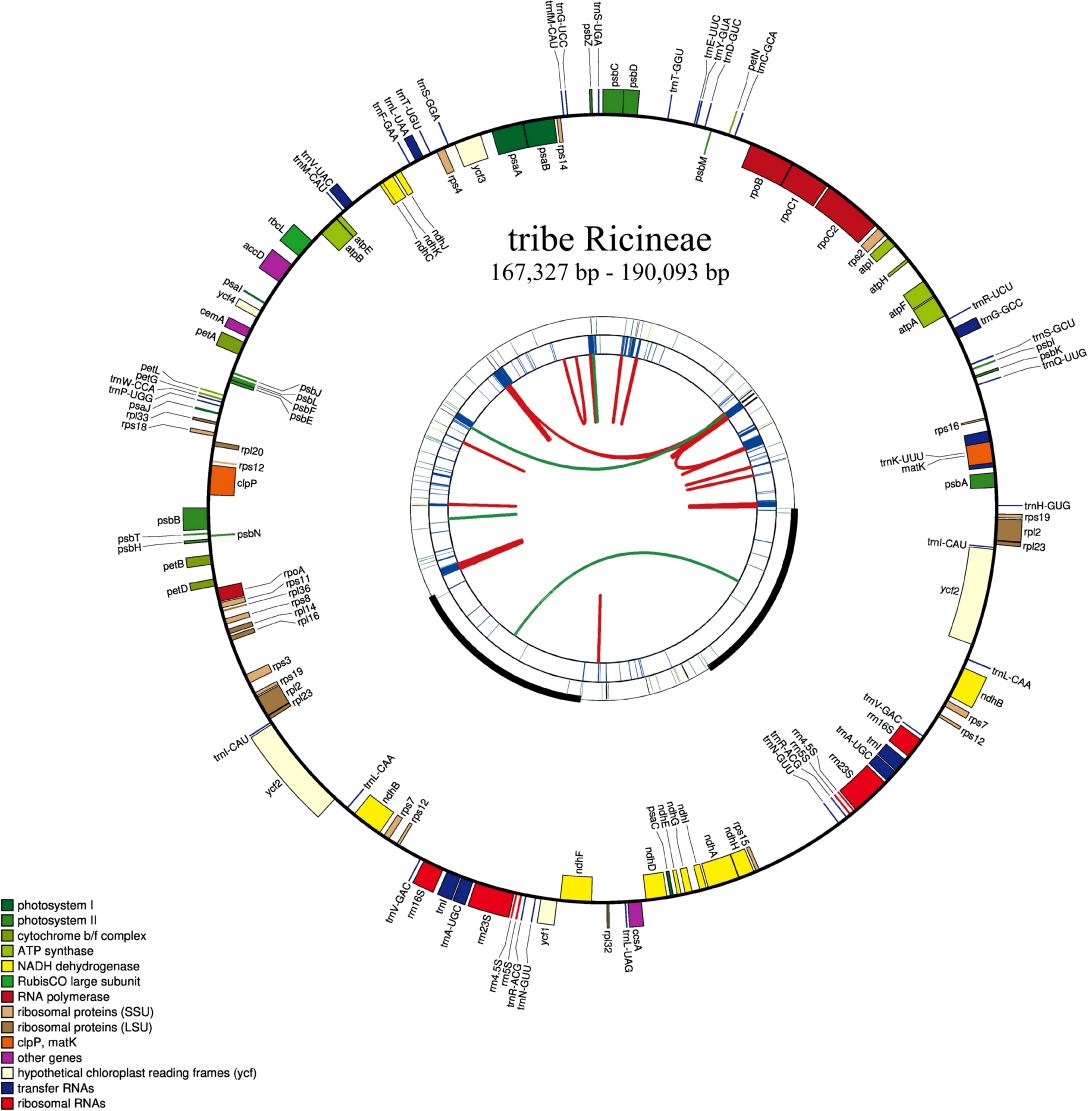
Plastome of a newly sequenced species of the tribe Ricineae. This map has four rings. Looking outward from the center, the first circle indicates forward and backward repeats connected by red and green arcs, respectively. The next circle is a tandem repetition marked with a short bar. The third circle is the sequence of microsatellites identified by MISA. The fourth circle was drawn using drawGenemap and represents the gene structure on the plastid. These genes are colored according to their functional class.

The plastomes of the five newly sequenced species were all typical circular quadripartite structure (**Figure 2**) and the length ranged from 167,327 bp to 190,093 bp. The GC content ranged from 31.02% to 34.93% (**Supplementary Table S4**). All the species contained 131 genes except *S. yunnanensis*, which had 130 genes (loss of the t*rnK-UUU* gene) (**Supplementary Table S5**).

**Figure 3:**
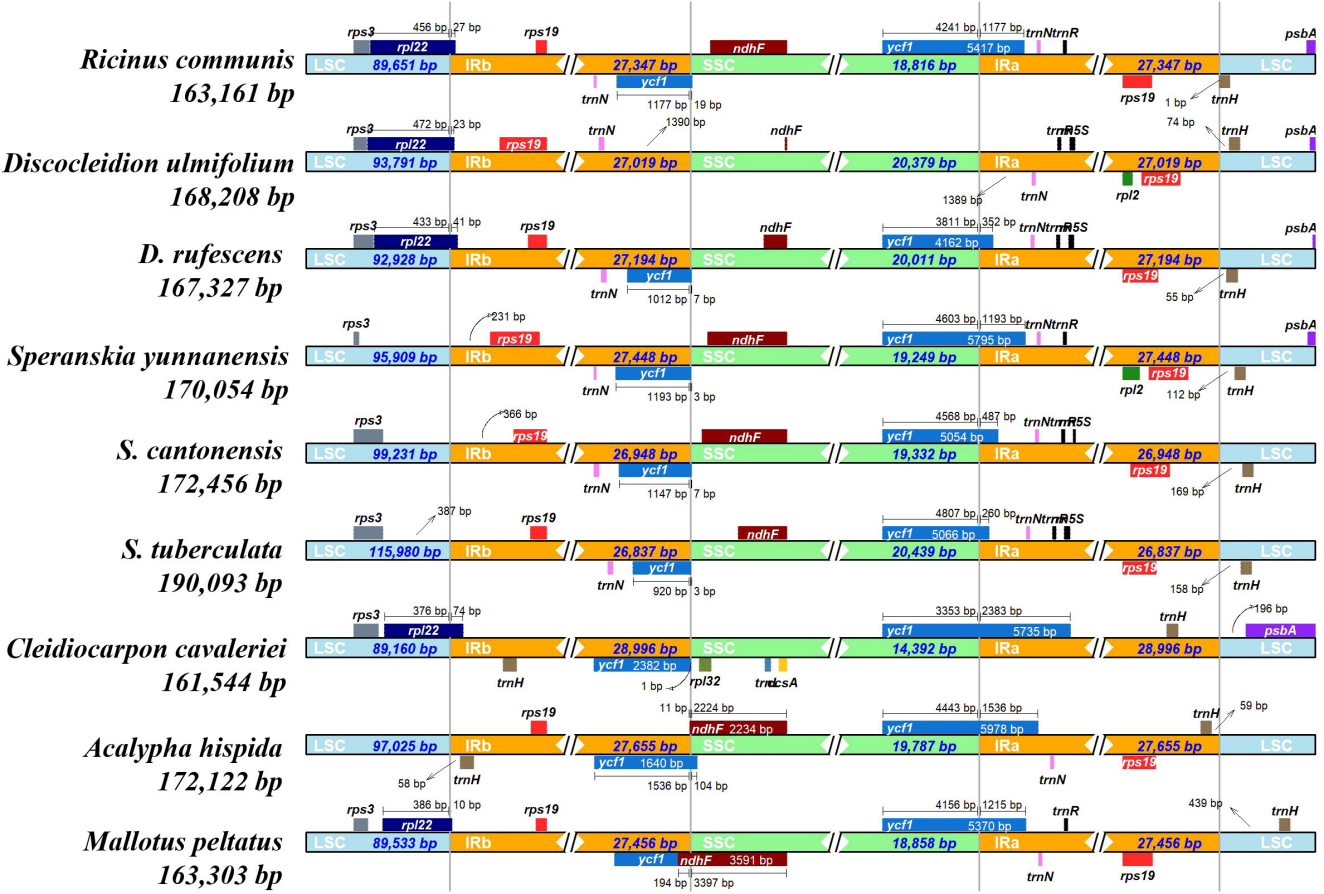
Structural variations in the plastomes of species in the tribe Ricineae and other species in the Euphorbiaceae. Genes transcribed forward are shown above, whereas genes transcribed reversely are shown below. Gene lengths in the corresponding regions are displayed above the boxes of gene names. The four grey lines denote the junction sites of LSC/IRb, SSC/IRb, SSC/IRa, and LSC/IRa, respectively. SSC, small single copy, IR, inverted repeats, LSC, large single copy.

Positive values for Tajima’s D (0.43) and Ka/Ks values which were ranging from 0.78 (between *D. ulmifolium* and *R. communis*) to 1.09 (between *D. rufescens* and *D. ulmifolium*) (**Supplementary Figure S1**) were calculated. The DNAsp5 showed 7,150 polymorphic sites, 7,438 mutations, and a Pi value of 0.02 for the six species. To further identify the regions with high variation, Pi analysis was performed on coding and non-coding regions. The results showed that there were 25 genes with a Pi value greater than 0.02 in the coding region (the highest value was located in *matK*, Pi=0.033) (**Supplementary Figure S2B**). In addition, there were 45 regions with a Pi value greater than 0.02 in the non-coding region (the highest value was located in *accD-psaI*, Pi=0.128) (**Supplementary Figure S2A**). The variations were mainly located in the large single copy (LSC) region, and there was a large gap between 90k and 93k, where the sequence similarity was less than 50%. The small single copy (SSC) region had the least variations, and most of the variations existed in the non-coding region (**Supplementary Figure S3A**). In the Subfam. Acalyphoideae, the sequence similarity in the 0–9k region was less than 50%, and the overall sequence similarity was relatively low (**Supplementary Figure S3B**). This indicated that the plastome of the tribe Ricineae is highly conserved and has undergone significant differentiation from its closely related groups. The IR region did not show significant contractions or expansions, and the boundary region was relatively conservative (**Figure 3**). The collinearity analysis indicated the six species showed high level of similarity and a good collinear relationship (**Supplementary Figure S4**).

Codon bias analysis showed that the CAI, CBI, Fop, and ENC ranged from 0.136 to 0.154, -0.166 to -0.109, 0.312 to 0.347, and 53.37 to 55.28, respectively (**Supplementary Table S6**). RSCU analysis revealed 61 synonymous codons (**Supplementary Figure S5**). Among the 31 codons with an RSCU value greater than one, 26 of them ended with A\T, while 24 of the 28 codons with an RSCU value smaller than one ended with G\C (**Supplementary Table S6**).

Long repetitive sequences with a length ≥ 30 bp and similarity > 90% between two copies were detected. In total, 1,253 long repetitive sequences were detected including 42–60 F repeats (34%), 21–36 R repeats (18%), 0–10 C repeats (4.8%), and 4–28 P repeats (63.2%) (**Supplementary Figure S6**). A total of 1,130 SSRs were found, including 60–91 single base repeats, 21–70 double base repeats, 5–70 triple base repeats, 19–62 quadruple base repeats, 4–37 quintuple base repeats, and 0–4 sextuple bases repeat sequences. *S. tuberculata* had the largest number of SSRs (n = 291), while *R. communis* had the smallest number of SSRs (n=144) (**Supplementary Figure S7**).

### 3.3 Phylogeny and divergence times

**Figure 4:**
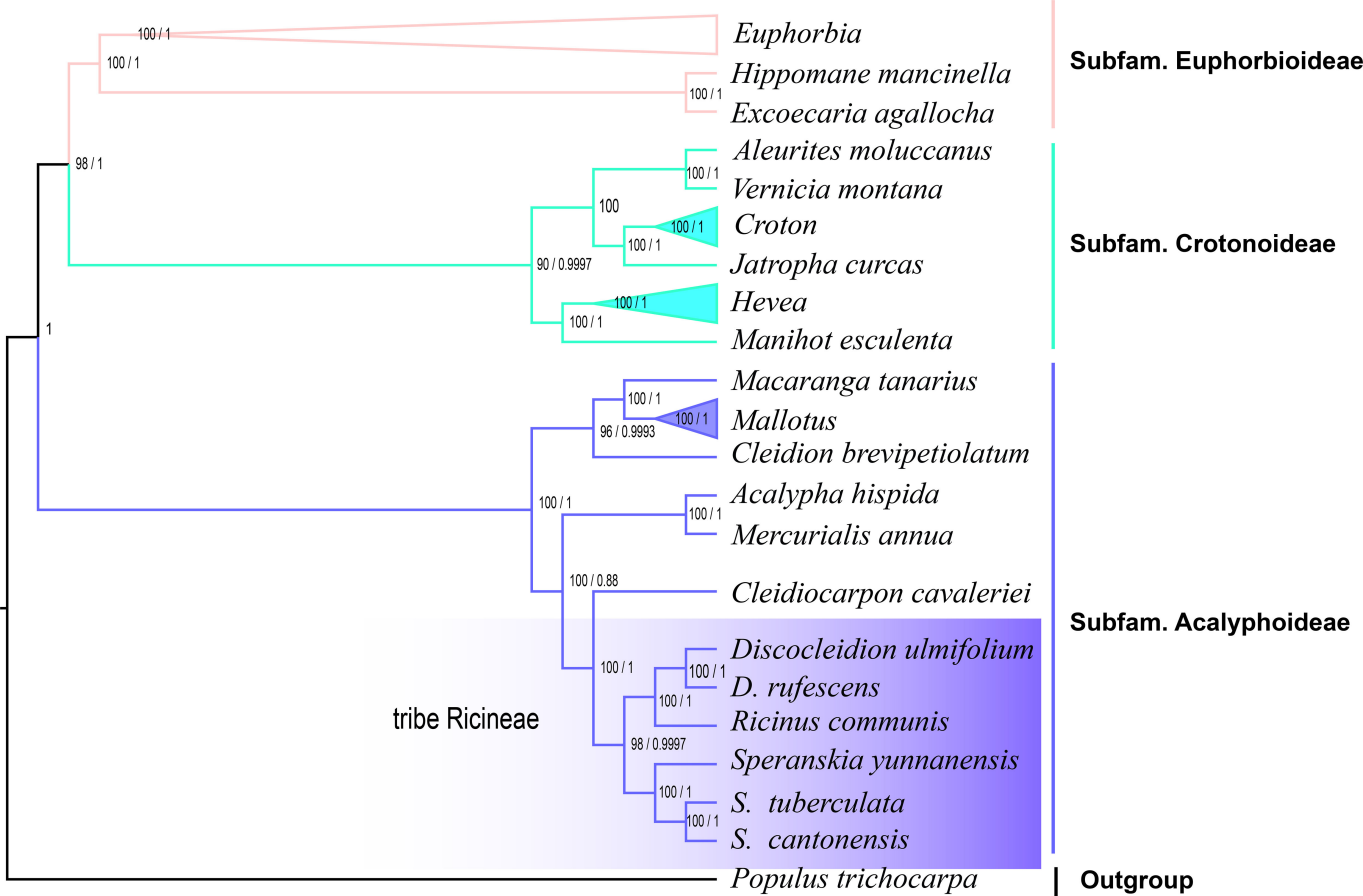
Phylogenetic trees of Euphorbiaceae reconstructed using maximum likelihood (ML) and Bayesian inference (BI) through plastomes. Bootstrap support (BS) values inferior to 100% and posterior probability to 1 are shown.

In the phylogenetic trees reconstructed by plastomes, three subfamilies, Subfam. Crotonoideae, Subfam. Euphorbioideae, and Subfam. Acalyphoideae (Sun et al., 2016), were revealed in both the ML and BI phylogenetic trees of Euphorbiaceae (**Figure 4**). The three genera formed a well supported (PP > 0.95) monophyletic lineage, which showed *Discocleidion* and *Ricinus* was the most closely related in the Subfam. Acalyphoideae in both trees. In the ML tree reconstructed using transcriptome data, the same monophyletic lineage that illustrates the same intergeneric and interspecific relationships was shown. All clades in the monophyletic lineage were well resolved (PP > 0.95, and bootstrap support, BS, > 90) in all the reconstructed phylogenetic trees.

**Figure 5:**
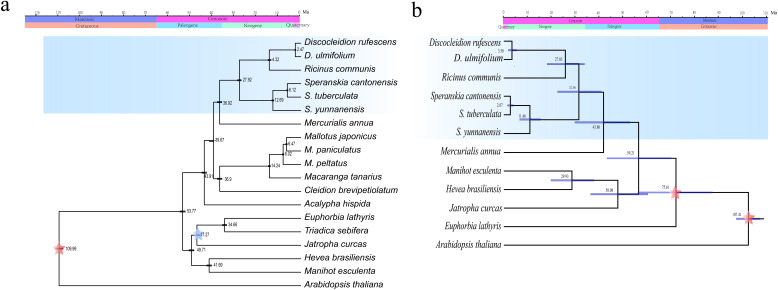
Divergence time estimation of the tribe Ricineae through plastome **(a)** or transcriptome **(b)** data. The fossil calibrations are labeled as blue asterisk and three secondary calibration points are labeled as red asterisk. Ma, million years ago.

Dating analysis indicated the crown age of the monophyletic lineage consisting of the three genera was estimated during the Oligocene-Miocene (27.92 and 32.96 Ma estimated by the plastome and transcriptome data, respectively) (**Figure 5**). The two East Asian endemic genera, *Speranskia* and *Discocleidion*, diversified in the late Miocene (12.69 or 11.46 Ma) and the Pliocene-Pleistocene (2.47 or 3.58 Ma), respectively.

### 3.4 Morphological analysis

In the FOC (Li, 1990), *Speranskia* is classified into the tribe. Chrozophoreae based on the presence or absence of petals in female flowers, while *Discocleidion* and *Ricinus* are classified into tribe Acalypheae, with a relatively simple classification criterion. However, through observations of specimens and living plants, and review of relevant materials, this study found that species belonging to these three genera share similar characteristics, including alternate leaves with serrated edges, terminal racemose or paniculate inflorescences with 3–5 calyx lobes arranged in a valvate manner, the absence of sterile pistils, a tricarpellary ovary with one ovule in each of the three chambers, and three bifid styles splitting into two lobes reaching the middle or almost to the base. Therefore, morphological evidence supports the classification of these three genera as being closely related.

The species in the tribe Ricineae exhibit significant morphological variations. To facilitate their identification, this study developed a dichotomous key based on traits such as monoecy/dioecy, leaf morphology, and floral characteristics.

Morphology can be used to distinguish the three genera of the tribe Ricineae well.

1. Flowers dioecious…………………………………………2
1. Flowers monoecious………………………………………3
2. Twigs, leaves, and inflorescences densely covered with white or pale yellow long soft hairs; fruits hairy……*D. rufescens*
2. Plant glabrous except for young shoots; fruits glabrous………………………………………*D. ulmifolium*
3. Male flowers without petals, leaf blade palmately 7-11-lobed…………………………………………..*R. communis*
3. Male flowers with petals, undivided leaves………………4
4. Female flowers with petals; petioles short, less than 5 mm long, fruits with tubercular protuberances….*S. tuberculata*
4. Female flowers without petals; petioles longer than 7 mm...5
5. Leaf tips acute or acuminate, margins with rounded or obtuse teeth; fruits with distinct tubercular protuberances………………………………...*S. cantonensis*
5. Leaf tips caudate-acuminate or caudate, margins with sharp teeth or serrations; fruits without tubercular protuberances………………………………..*S. yunnanensis*

## 4 Discussion

### 4.1 Intergeneric and interspecific relationships in the tribe Ricineae

Although *Speranskia*, and *Discocleidion* are likely closely related genera to *Ricinus*, the intergeneric relationship of the tribe *Ricineae* is inconsistent using different genes (Sun et al., 2016; Zhou et al., 2017; Zuntini et al., 2024). The intergeneric relationship was reassessed through the phylogenomic analyses of plastome and transcriptome, supplemented by a morphological investigation, in this study.

The monophyletic lineage (*Ricinus*, *Speranskia*, and *Discocleidion*) revealed by previous molecular studies (Sun et al., 2016; Zhou et al., 2017) through DNA sequence fragments were further consistently verified using different genomic data (the plastome and transcriptome) using different methods (BI and ML) for phylogenetic tree reconstructions. The same intergeneric and interspecific relationships of the lineage are also well supported (PP > 0.95 and BS > 90). *Speranskia* form a clade that is sister to the clade including *Ricinus* and *Discocleidion*, and in *Speranskia, S. tuberculata* and *S. cantonensis* are most closely related sister species. Based on the phylogenetic evidence and morphological similarities, such as alternate leaves with serrated edges, racemose or paniculate inflorescences that are terminal, and so on, we propose the most closely related genera of *Ricinus* are *Speranskia* and *Discocleidion*. Furthermore, in Euphorbiaceae, both the ML and BI trees reconstructed through plastome support the classification of three subfamilies, Crotonoideae, Euphorbioideae, and Acalyphoideae.

Due to the plasticity of morphological characteristics and parallel evolution, the morphological classification of Euphorbiaceae primarily relies on a limited number of traits such as seed surface features (Frajman and Schönswetter, 2011). Furthermore, the chromosome number also poses significant challenges for effective classification (Hans, 1973). Therefore, conflicts between morphological classification and phylogenetic analyses are relatively common in Euphorbiaceae. For example, *Dimorphocalyx* and *Tritaxis* are classified under *Trigonostemon* based on morphological features (Webster, 2014), but molecular phylogenetic analysis shows they form an independent monophyletic group, differing from traditional classifications. Molecular evidence indicates that *Euphorbia tuckeyana* is a sister group to sect. *Aphyllis* (Barres et al., 2011), but it is still included in subsect. *Macaronesicae* based on morphological similarities (Riina et al., 2013).

The crown age of the tribe is estimated to be Oligocene-Miocene, similar to Yuan et al. (2022) who showed the coalescence of the tribe at 26.43 Ma based on nuclear genomes, which is likely correlated to the origin of the East Asian monsoon (Ye et al., 2022). Afterward, the diversification of the two East Asian endemic genera is likely correlated with intensification of the monsoon, mountain uplift during the Miocene-Pliocene (*Speranskia*) (Xing and Ree, 2017), and glacial-interglacial alternations, accompanied by sea level changes during the Pleistocene (*Discocleidion*) (Qiu et al., 2011). *Speranskia* is likely originated in southwest China (*S. yunnanensis*) and the other two species diverge due to Pleistocene climatic changes (Qiu et al., 2011).

### 4.2 Structure of plastome and comparative analyses

The plastome sizes of the species in the tribe Ricineae range from 163,161 (*R. communis*) to 190,093 bp (*S. tuberculata*), all of which have a typical circular quartet structure. *S. tuberculata* has the longest genome size in the tribe Ricineae, which may mainly due to expansions of the non-coding region of LSC and a higher number of SSRs (quadruple and quintuple base SSRs). The IR regions show no significant variations (Braukmann et al., 2013).

SSRs play a crucial role in stabilizing genomes and rearranging genomic sequences, thereby rendering the plastome highly favorable for use as a molecular marker in phylogenetic analysis (Vieira et al., 2014; Yang et al., 2013). This study detects 144–291 SSRs in the six species of the tribe Ricineae, which is generally higher than other species within the Euphorbiaceae. Among the dinucleotide SSRs, AT is found to be the most abundant in the sequenced and compared plastomes, similar to the previously reported genomes (Khan et al., 2020, Khan et al., 2017). The Pi of the tribe Ricineae (Pi=0.02) is relatively high compared to other Euphorbiaceae species (Lee et al., 2024; Wang et al., 2024), and regions with high diversity such as *matK*, *ndhF*, *psbT*, *rpl20*, and *rpl32* are potential areas for designing DNA barcodes and allele-specific primers.

Since the selective pressure on the third base of codons is relatively weaker compared to the first and second bases, a comparison of the base composition of the third base of codons is important (Zhang et al., 2018). The probability of A/T as the third base composition in the tribe Ricineae ranges from 82.1% to 88.9%, and among the high-frequency codons with an RSCU greater than one (**Supplementary Figure S5**), the probability of ending with A/T is 83.9%, which is consistent with previous studies on codon bias in related species of Euphorbiaceae (Novembre, 2002; Wang et al., 2020). Prior research has shown that mutation pressure and natural selection are the primary factors influencing codon bias (Sharp et al., 2010). However, combining the conclusions of Wang et al. (2020) regarding codon bias in other species of Euphorbiaceae and the fact that the ka/ks ratio is close to one, it can be inferred that natural selection is the main factor influencing codon usage bias in the tribe Ricineae. The relatively low level of the codon bias suggests that the natural selection pressure on chloroplast genes in these species is relatively small. A positive value in Tajima’s D test also indicates a tendency toward population contraction.

## 5 Conclusion

The intergeneric and interspecific relationships of the tribe Ricineae are reassessed through a combination of different genomic and morphological data. The tribe fromed a well-supported monophyletic lineage and *Discocleidion* is the closest affiliate to *Ricinus*, providing a genetic base for future comparative genomic studies. The similarities and dissimilarities of the plastomes in the tribe provide further information for the phylogentic investigation of Euphorbiaceae. Phylogeny reconstruction of closely related genera through different genomes provides a case study for solving the complications of Euphorbiaceae taxonomy.

## Data Availability

The datasets presented in this study can be found in online repositories. The names of the repository/repositories and accession number(s) can be found below: https://www.ncbi.nlm.nih.gov/genbank/, PP941955 https://www.ncbi.nlm.nih.gov/genbank/, PP941956 https://www.ncbi.nlm.nih.gov/genbank/, PP941957 https://www.ncbi.nlm.nih.gov/genbank/, PP968730 https://www.ncbi.nlm.nih.gov/genbank/, PQ009256.
