# Supplementary material for: Intergeneric and interspecific relationships in tribe Ricineae revealed by phylogenomics of the plastome and transcriptome
Source: Front Plant Sci. 2025 May 1;16:1544247. doi: 10.3389/fpls.2025.1544247 (PMC12078312; doi:10.3389/fpls.2025.1544247)
Supplement: Supplementary file 1 [file DataSheet1.docx]

**Supplementary material**

**Intergeneric and interspecific relationships in tribe Ricineae revealed by phylogenomics of the plastome and transcriptome**

Wen-Xiang Liu, Guo-Bo Li, Zhuo Zhou, Jia-Fu Chen, An-Min Yu, Ai-Zhong Liu, Bin Tian and Jun-Wei Ye

**Supplementary Table 1** Sample collection information.

| **Species** | **Collecting locations** | **Coordinate** |
| --- | --- | --- |
| *Speranskia tuberculata* | Shizhong County, Shandong Province, China | 35.5655N, 36.6216E |
| *S.* *cantonensis* | Ruyuan County, Gangdong Province, China | 24.9911N, 113.1416E |
| *S. yunnanensis* | Menglian County, Yunnan Province, China | 22.3322N, 99.5803E |
| *Discocleidion rufescens* | Yanshan County, Guangxi Province, China | 25.0825,N 110.2933E |
| *D. ulmifolium* | Qingtian County, Zhejiang Province, China | 28.3040N, 119.9727E |

**Supplementary Table 2** Accession numbers for transcriptome data in Genbank.

| **Species** | **Accession number** |
| --- | --- |
| *Ricinus communis* | PRJNA838012 |
| *Jatropha curcas* | PRJNA673911 |
| *Euphorbia lathyris* | PRJNA1119998 |
| *Hevea brasiliensis* | PRJNA976717 |
| *Manihot esculenta* | PRJNA394209 |
| *Mercurialis annua* | PRJNA872570 |
| *Arabidopsis thaliana* | PRJNA10719 |

**Supplementary Table 3** Sequencing and assembly statistics for transcriptome of the six species used in this study.

| **Species** | **Genes number** | **GC content (%)** | **BUSCO**  **(%)** | **Contig N50（bp）** | **Average contig（bp）** |
| --- | --- | --- | --- | --- | --- |
| *Speranskia tuberculata* | 58866 | 38.54 | 94.6 | 2489 | 1341.13 |
| *S. cantonensis* | 50513 | 38.17 | 95.2 | 2385 | 966 |
| *S. yunnanensis* | 60214 | 38.20 | 96.9 | 2313 | 1396.46 |
| *Discocleidion rufescens* | 76309 | 39.94 | 93 | 1506 | 802.42 |
| *D. ulmifolium* | 78048 | 39.37 | 96.3 | 2149 | 1062.77 |
| *Ricinus communis* | 67361 | 40.01 | 99.5 | 2239 | 1923.10 |

**Supplementary Table 4** Basic information of plastome of five newly sequenced species of the tribe Ricineae. SSC, small single copy, IR, inverted repeats, LSC, large single copy.

| **Species** | **Length (bp)** | | | |  | | **GC content (%)** | | | |
| --- | --- | --- | --- | --- | --- | --- | --- | --- | --- | --- |
|  | **Total** | **LSC** | **SSC** | **IR** | |  | **Total** | **LSC** | **SSC** | **IR** |
| *Speranskia tuberculata* | 190093 | 115981 | 20440 | 26836 | |  | 31.02 | 26.31 | 28.36 | 42.20 |
| *S. cantonensis* | 172459 | 99232 | 19333 | 26947 | |  | 34.20 | 30.65 | 29.99 | 42.25 |
| *S. yunnanensis* | 170054 | 95910 | 19250 | 27447 | |  | 34.58 | 31.51 | 29.74 | 41.64 |
| *Discocleidion rufescens* | 167327 | 92929 | 20012 | 27193 | |  | 34.93 | 32.28 | 28.00 | 42.00 |
| *D. ulmifolium* | 168208 | 93792 | 20380 | 27018 | |  | 34.93 | 32.17 | 29.29 | 41.63 |

**Supplementary Table** **5** Number of annotated genes in plastome of five newly sequenced species in the tribe Ricineae.

| **Species** | **CDS (Coding sequence)** | **tRNA** | **rRNA** | **Total** |
| --- | --- | --- | --- | --- |
| *Speranskia tuberculata* | 86 | 37 | 8 | 131 |
| *S. cantonensis* | 86 | 37 | 8 | 131 |
| *S. yunnanensis* | 86 | 36 | 8 | 130 |
| *Discocleidion rufescens* | 86 | 37 | 8 | 131 |
| *D. ulmifolium* | 86 | 37 | 8 | 131 |

**Supplementary Table** **6** Codon bias of plastome of the six species in the tribe Ricineae. CAI, codon adaptation index, CBI, codon bias index, ENC, effective number of codons, Fop, frequency of optimal codons.

| **Species** | **T3s** | **C3s** | **A3s** | **G3s** | **CAI** | **CBI** | **Fop** | **ENC** |
| --- | --- | --- | --- | --- | --- | --- | --- | --- |
| *Speranskia tuberculata* | 0.4456 | 0.1964 | 0.4434 | 0.2210 | 0.136 | -0.166 | 0.312 | 53.37 |
| *S. cantonensis* | 0.4193 | 0.2255 | 0.4156 | 0.2361 | 0.150 | -0.118 | 0.340 | 55.06 |
| *S. yunnanensis* | 0.4143 | 0.2344 | 0.4069 | 0.2368 | 0.149 | -0.115 | 0.344 | 55.28 |
| *Discocleidion rufescens* | 0.4238 | 0.2255 | 0.4155 | 0.2242 | 0.154 | -0.112 | 0.345 | 54.68 |
| *D. ulmifolium* | 0.4182 | 0.2329 | 0.4063 | 0.2379 | 0.154 | -0.109 | 0.347 | 55.14 |
| *Ricinus communis* | 0.4203 | 0.2223 | 0.4167 | 0.2220 | 0.154 | -0.113 | 0.345 | 54.43 |

**Supplementary Table** **7** Accession numbers of downloaded plastomes in reconstructing phylogenetic tree of Euphorbiaceae.

| **Species** | **GenBank accession number** | **Species** | **GenBank accession number** |
| --- | --- | --- | --- |
| *Euphorbia adenochlora* | OR400574.1 | *Euphorbia orobanchoides* | MT395043.1 |
| *Euphorbia alluaudii* | MT395034.1 | *Euphorbia pekinensis* | MZ707776.1 |
| *Euphorbia altotibetica* | OP032235.1 | *Euphorbia pereskiifolia* | MT395004.1 |
| *Euphorbia ampliphylla* | MT395036.1 | *Euphorbia poissonii* | MT395035.1 |
| *Euphorbia bicompacta* | MT395030.1 | *Euphorbia prostrata* | OQ184029.1 |
| *Euphorbia biselegans* | MT395022.1 | *Euphorbia pseudolaevis* | MT395047.1 |
| *Euphorbia bisglobosa* | MT395041.1 | *Euphorbia pseudomollis* | MT528642.1 |
| *Euphorbia brunellii* | MT528638.1 | *Euphorbia pseudonudicaulis* | MT528643.1 |
| *Euphorbia catenata* | MT395044.1 | *Euphorbia pseudosimplex* | MT528648.1 |
| *Euphorbia chevalieri* | MT528649.1 | *Euphorbia pteroneura* | MW496386.1 |
| *Euphorbia crotonoides* | MW496380.1 | *Euphorbia pudibunda* | MT528640.1 |
| *Euphorbia cupricola* | MW300677.1 | *Euphorbia renneyi* | MT394999.1 |
| *Euphorbia discoidea* | MT395042.1 | *Euphorbia rhizophora* | MT395020.1 |
| *Euphorbia drupifera* | MW496383.1 | *Euphorbia ritchiei* | MT394998.1 |
| *Euphorbia ebracteolata* | MT830860.1 | *Euphorbia royleana* | OQ397538.1 |
| *Euphorbia echinulata* | MT395011.1 | *Euphorbia rubella* | MT528639.1 |
| *Euphorbia enterophora* | MT395033.1 | *Euphorbia scheffleri* | MT395025.1 |
| *Euphorbia espinosa* | MW496384.1 | *Euphorbia schlechtendalii* | MW496378.1 |
| *Euphorbia esula* | KY000001.1 | *Euphorbia schubei* | MT395017.1 |
| *Euphorbia fauriei* | OP477346.1 | *Euphorbia smithii* | MN646684.1 |
| *Euphorbia fwambensis* | MT528641.1 | *Euphorbia succulenta* | MT395015.1 |
| *Euphorbia guentheri* | MT395002.1 | *Euphorbia syncameronii* | MT395039.1 |
| *Euphorbia hedyotoides* | MT395028.1 | *Euphorbia thymifolia* | OQ184030.1 |
| *Euphorbia helioscopia* | OP169347.1 | *Euphorbia tirucalli* | MH890571.1 |
| *Euphorbia hirta* | MW429224.1 | *Euphorbia tithymaloides* | MW496382.1 |
| *Euphorbia humifusa* | OR189520.1 | *Euphorbia torrei* | MT395013.1 |
| *Euphorbia hypericifolia* | OQ184033.1 | *Euphorbia umbellata* | MT395046.1 |
| *Euphorbia invenusta* | MT395005.1 | *Euphorbia yattana* | MT395024.1 |
| *Euphorbia jolkinii* | OR400590.1 | *Hippomane mancinella* | MW255977.1 |
| *Euphorbia kansui* | MH392274.1 | *Euphorbia peplus* | MZ678242.1 |
| *Euphorbia kirkii* | MW300679.1 | *Aleurites moluccanus* | MW322810.1 |
| *Euphorbia laric* | MN646683.1 | *Croton laevigatus* | MN713923.1 |
| *Euphorbia lathyris* | MT830859.1 | *Croton tiglium* | MH394334.1 |
| *Euphorbia lindenii* | MT395026.1 | *Croton yunnanensis* | OM630154.1 |
| *Euphorbia lugardae* | MT395019.1 | *Excoecaria agallocha* | MZ687828.1 |
| *Euphorbia maculata* | OR189521.1 | *Hevea benthamiana* | MT333859.1 |
| *Euphorbia mafingensis* | MW300676.1 | *Hevea brasiliensis* | NC015308.1 |
| *Euphorbia magnifica* | MT395016.1 | *Hevea camargoana* | MN781109.1 |
| *Euphorbia major* | MT528646.1 | *Hevea nitida* | MT413435.1 |
| *Euphorbia makinoi* | OQ184031.1 | *Hevea pauciflora* | MW528030.1 |
| *Euphorbia mbuinzauensis* | MT395000.1 | *Hevea spruceana* | MW528031.1 |
| *Euphorbia milii* | MW496385.1 | *Jatropha curcas* | NC012224.1 |
| *Euphorbia neoarborescens* | MT395032.1 | *Manihot esculenta* | NC010433.1 |
| *Euphorbia neocrispa* | MT528647.1 | *Vernicia montana* | MW297080.1 |
| *Euphorbia neocymosa* | MT395006.1 | *Acalypha hispida* | OP546127.1 |
| *Euphorbia neoglabrata* | MT394996.1 | *Cleidiocarpon cavaleriei* | MG813873.1 |
| *Euphorbia neoglaucescen* | MT395038.1 | *Macaranga tanarius* | MW297079.2 |
| *Euphorbia neogossweileri* | MT395027.1 | *Mallotus japonicus* | MW244068.1 |
| *Euphorbia neokaessneri* | MT535856.1 | *Mallotus paniculatus* | MZ597547.1 |
| *Euphorbia neoparviflora* | MT528644.1 | *Cleidiocarpon cavaleriei* | NC063572.1 |
| *Euphorbia neorubella* | MT395003.1 | *Cleidion brevipetiolatum* | OL804290.1 |
| *Euphorbia neospinescens* | MT259040.1 | *Mallotus peltatus* | MN885802.1 |
| *Euphorbia neostolonifera* | MT395023.1 | *Mercurialis annua* | OX359234.1 |
| *Euphorbia neovirgata* | MT395018.1 | *Ricinus communis* | JF937588.1 |
| *Euphorbia nutans* | OQ871366.1 | *Populus trichocarpa* | EF489041.1 |
| *Arabidopsis thaliana* | NC000932.1 | *Triadica sebifera* | MT424756.1 |

**Supplementary Table** **8** The genes of plastome of species in the tribe Ricineae.

| **Gene classification** | **Gene group** | **The name of the gene** | |
| --- | --- | --- | --- |
| Gene related to photosynthesis | photosystem Ⅰ | *psa*A *psa*B *psa*C *psa*I *psa*J | |
|  | photosystem II | *psb*A *psb*B *psb*C *psb*D *psb*E *psb*F *psb*H *psb*I *psb*J *psb*K *psb*L *psb*M *psb*N *psb*T *psb*Z | |
|  | cytochrome b/f complex | *pet*A *pet*B *pet*D *pet*G *pet*L *pet*N | |
|  | ATP synthase | *atp*A *atp*B *atp*E *atp*F *atp*H *atp*I | |
|  | NADH dehydrogenase | *ndh*A *ndh*B *ndh*C *ndh*D *ndh*E *ndh*F *ndh*G *ndh*H *ndh*I *ndh*J *ndh*K | |
|  | rubisCO large subunit | *rbc*L | |
| Expression of related genes | ribosomal proteins (LSU) | *rpl2 rpl14 rpl16 rpl20 rpl22 rpl23 rpl32 rpl33 rpl36* | |
|  | ribosomal proteins (SSU) | *rps*2 *rps*3 *rps*4 *rps*7 *rps*8 *rps*11 *rps*12 *rps*14 *rps*15 *rps*16 *rps*18 *rps*19 | |
|  | transfer RNAs | *trn*A-UGC *trn*C-GCA *trn*D-GUC *trn*E-UUC *trn*F-GAA *trnf*M-CAU *trn*G-GCC *trn*G-UCC *trn*H-GUG *trn*I-CAU *trn*I-GAU *trn*K-UUU *trn*L-CAA *trn*L-UAA *trn*L-UAG *trn*M-CAU *trn*N-GUU *trn*P-UGG *trn*Q-UUG *trn*R-ACG *trn*R-UCU *trn*S-GCU *trn*S-GGA *trn*S-UGA *trn*T-GGU *trn*T-UGU *trn*V-GAC *trn*V-UAC *trn*W-CCA *trn*Y-GUA |  |
|  | RNA polymerase | *rpo*A *rpo*B *rpo*C1 *rpo*C2 | |
|  | ribosomal RNAs | *rrn*4.5 *rrn*5 *rrn*16 *rrn*23 | |
| Other genes |  | *ccs*A *acc*D *cem*A *clp* P *mat* K | |
| Hypothetical chloroplast reading frames | | *ycf*1 *ycf*2 *ycf3 ycf4* | |


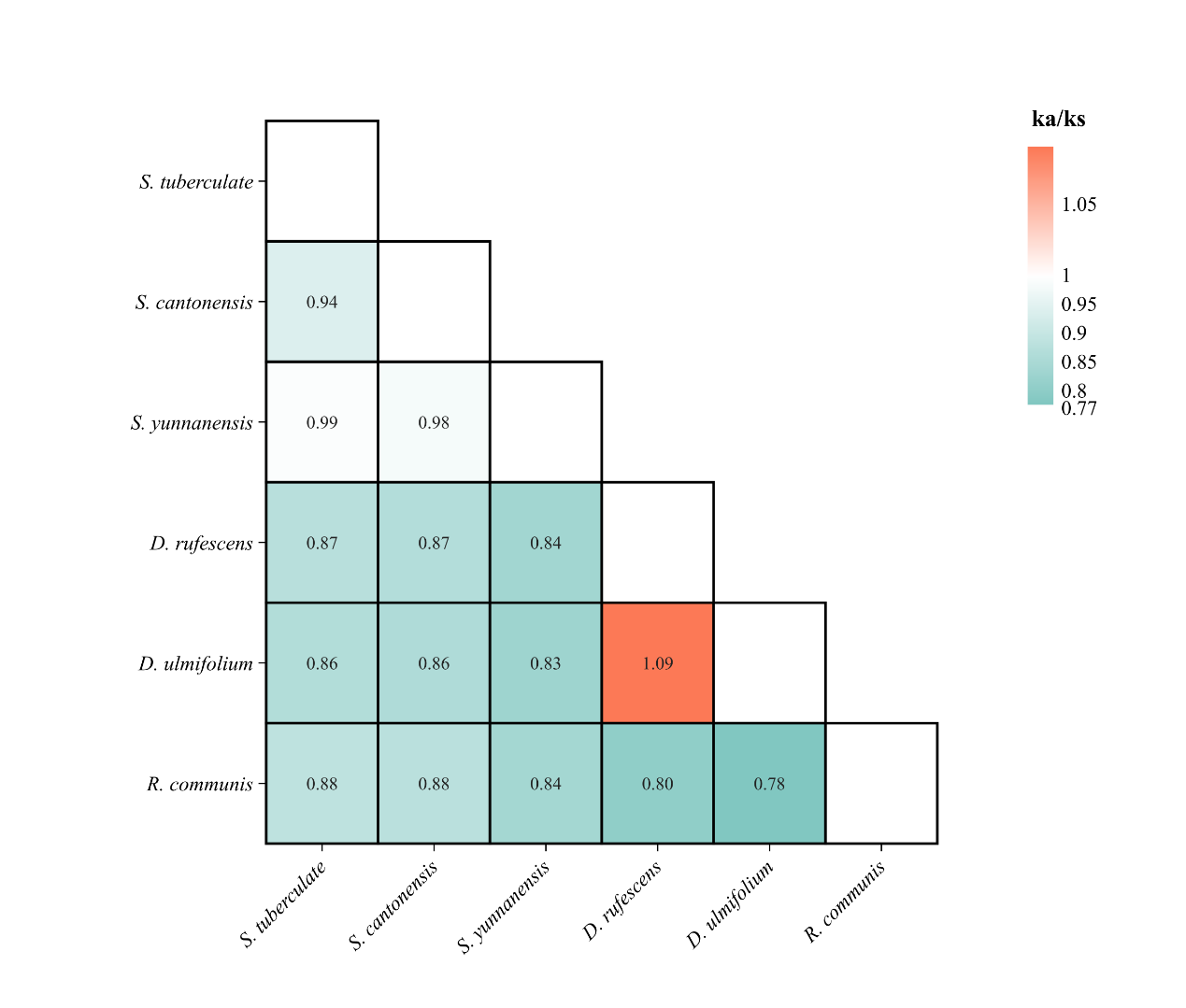


**Supplementary Figure S1** Ka/Ks ratios of plastome of the six species in the tribe Ricineae (*Speranskia*, *Discocleidion* and *Ricinus*).


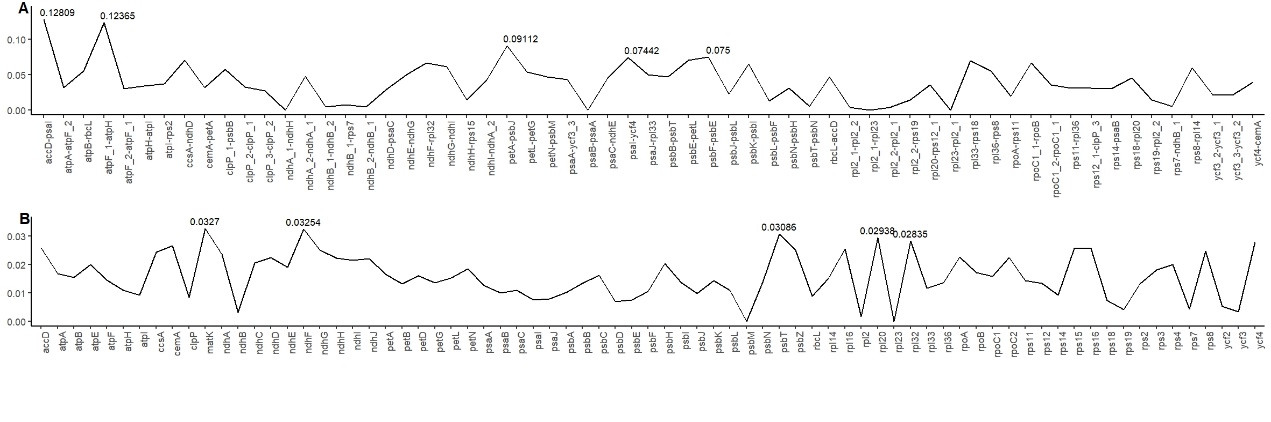


**Supplementary Figure S2** Nucleotide diversity (Pi) evaluation of the tribe Ricineae in the non-coding region (A) and coding region (B).


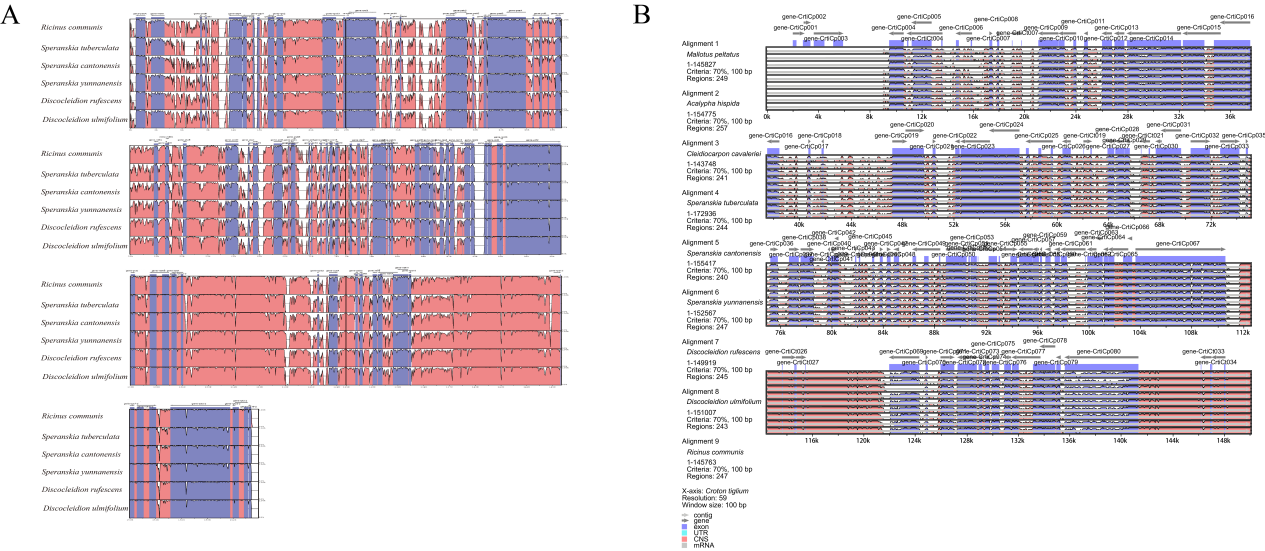


**Supplementary Figure S3** Comparison of plastome of species in the tribe Ricineae (A) and in the Subfam. Acalyphoideae (B). The y-axis represents the percent identity spanning 50-100%. Different regions are color-coded as coding region (purple), non-coding sequences (red).


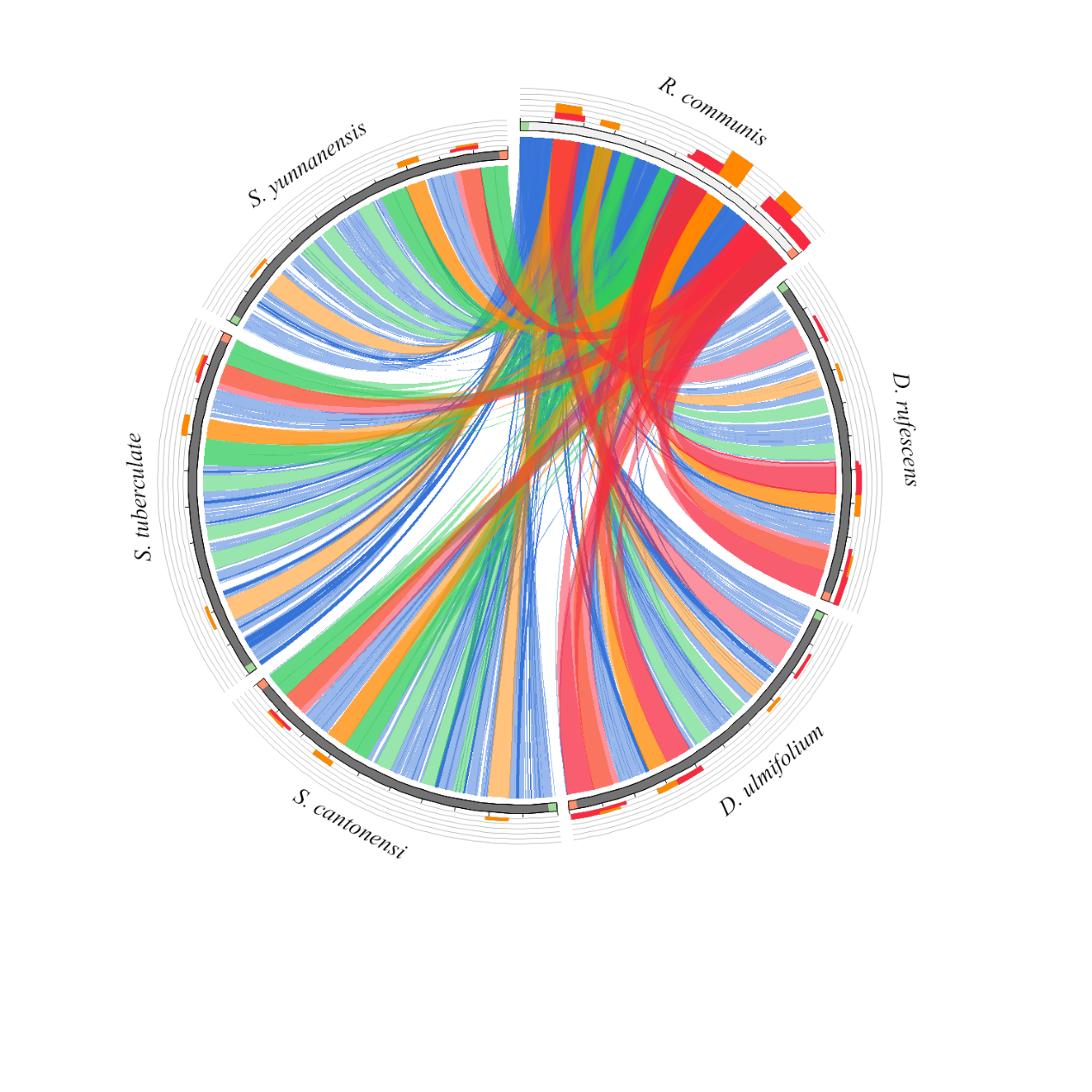


**Supplementary Figure S4** Collinearity analysis of the six species in the tribe Ricineae (*Speranskia*, *Discocleidion* and *Ricinus*). Blue, green, orange and red color represent 0-25%, 25-50%, 50-75% and 75-100% of the highest score, respectively.


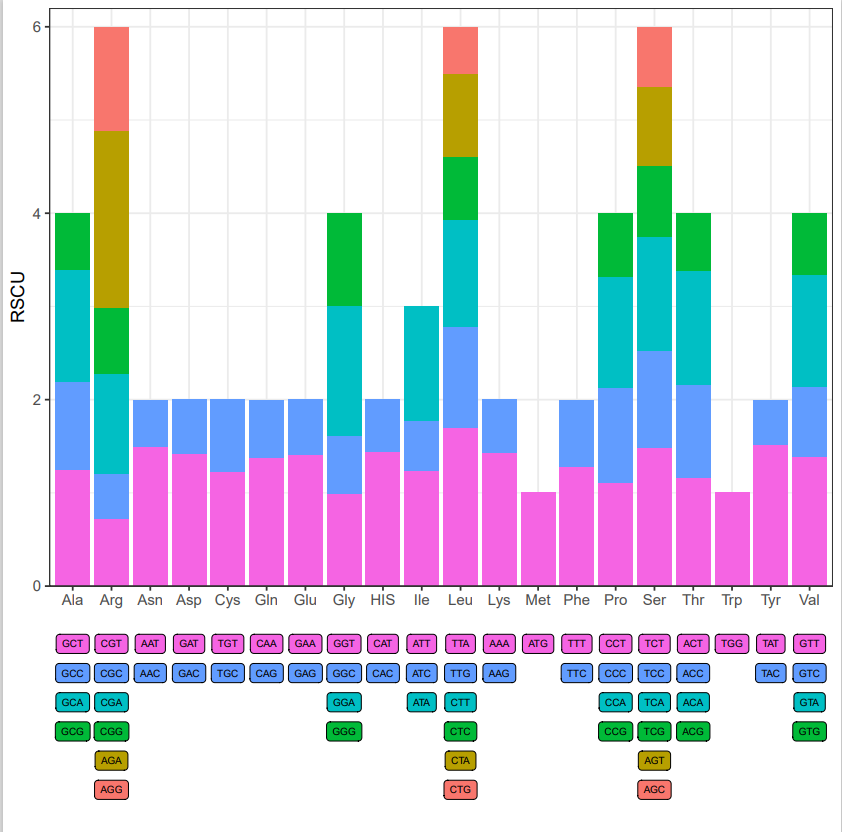


**Supplementary Figure S5** The relative synonymous codon usage (RSCU) of plastome of the six species in the tribe Ricineae.


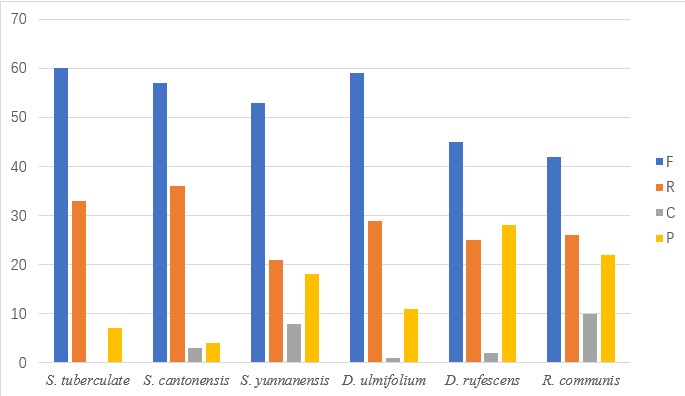


**Supplementary Figure S6** Frequency of four long repeat types: Forward (F), Reverse (R), Complement (C), Palindromic (P) in plastomes of six species in the tribe Ricineae (*Speranskia*, *Discocleidion* and *Ricinus*).


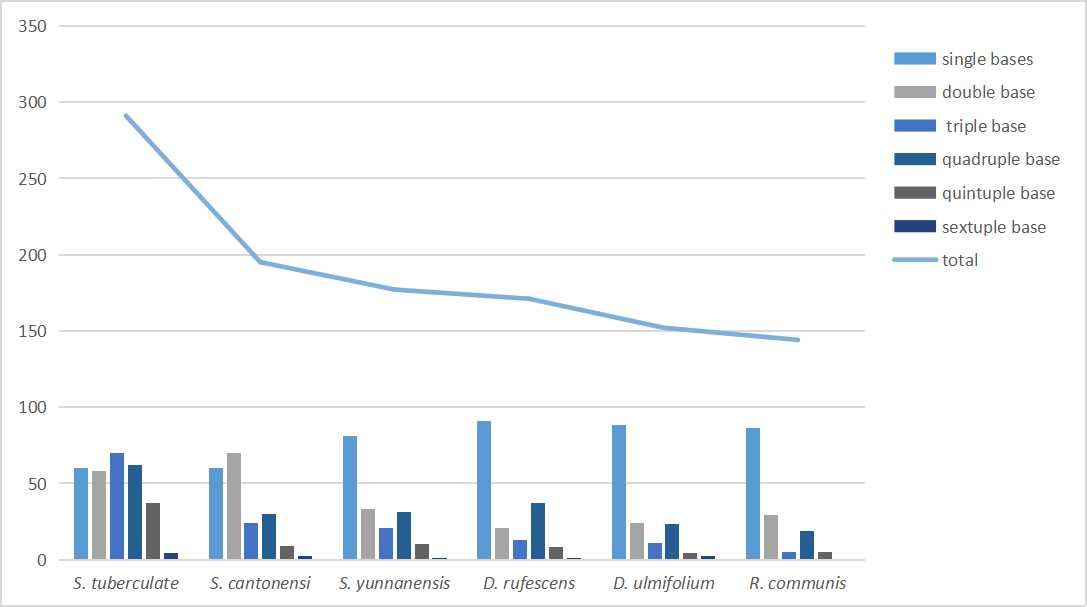


**Supplementary Figure S6** Frequency of six types of simple sequence repeats (SSRs) in plastomes of six species in the tribe Ricineae (*Speranskia*, *Discocleidion* and *Ricinus*).
